# Panorama e Estratégias no Diagnóstico e Tratamento de Cardiopatias Congênitas no Brasil

**DOI:** 10.36660/abc.20200680

**Published:** 2020-12-01

**Authors:** Fabio Augusto Selig

**Affiliations:** CONCEPT CuritibaPR Brasil CONCEPT - Centro de Cardiopatias Congênitas e Estruturais do Paraná, Curitiba, PR - Brasil

**Keywords:** Constituição e Estatutos, Sistema Único de Saúde, Cardiopatias Congênitas/diagnóstico, Financiamento de Assistência à Saúde

A Constituição Brasileira de 1988, também chamada de “Constituição Cidadã”, estabeleceu a universalização de acesso à saúde quando em seu artigo 196 colocou que esta é um direito de todos e dever do Estado. Pouco mais de uma década depois foi criado o Sistema Único de Saúde (SUS), que para respeitar os princípios de universalidade, integralidade e igualdade firmados na Carta Magna acrescentou diretrizes de descentralização, atendimento integral e participação popular.

Em muito inspirado à época no sistema de saúde inglês, o SUS sem dúvida é um dos maiores sistemas públicos de saúde do mundo, atendendo cerca de 190 milhões de pessoas, sendo que 80% destas dependem exclusivamente dele para tratar da sua saúde, por não participarem do sistema privado. Ainda, mesmo dentre os participantes do sistema privado é garantido constitucionalmente o uso do sistema público sem limites ou restrições.

O financiamento do SUS é sem dúvida um dos maiores desafios enfrentados nos seus 20 anos de existência, pois apesar dos recursos serem, em tese, garantidos pela Emenda Constitucional 29 e por vários outros dispositivos legais, a amplitude de sua cobertura torna inviável a atualização constante de procedimentos cobertos e valores pagos. Aliás, apesar de ser, em tese, universal e integral, a consequência da dificuldade de financiamento é o sistema público oferecer recursos preventivos, diagnósticos e terapêuticos menos abrangentes que no sistema privado, criando-se com o passar do tempo um abismo entre os dois. Torna-se essencial desta forma a criação de políticas claras de saúde pública para administrar estes recursos, e provavelmente em algum momento uma ampla rediscussão do papel dos sistemas público e privado no atendimento à população.

Nesta edição teremos a oportunidade de ler um importante artigo.^1^ De maneira clara, objetiva e técnica, ele demonstra com números concretos obtidos do banco de dados oficial do SUS a importância que as cardiopatias congênitas têm na mortalidade de jovens abaixo de 20 anos de idade, e em especial nas crianças abaixo de 1 ano. Podemos ver pelo levantamento apresentado que esta cobertura universal não encontra reflexo na vida real quando se trata deste grupo de doenças.

A incidência de cardiopatias congênitas na população em geral é de 8 em cada 1.000 nascidos vivos.^2^ Utilizando-se a taxa de natalidade brasileira,^3^ teríamos aproximadamente 30.000 novos nascimentos de crianças portadoras de doenças cardíacas. Em pelo menos metade destes recém-nascidos a cardiopatia apresenta boa evolução, com cura espontânea ou sem gravidade ao ponto do diagnóstico ser realizado apenas na idade adulta.^4^ Portanto, ainda de forma aproximada, teríamos 15.000 recém natos que anualmente necessitariam de algum tipo de tratamento, muitas vezes cirúrgico ou pela cardiologia intervencionista, e muitos logo após o nascimento ou no primeiro ano de vida.

Mantendo este raciocínio, dados oficiais apontam o escasso número de hospitais de alta complexidade cardiovascular que prestam atendimento ao SUS no Brasil.^5^ A Política Nacional de Atenção Cardiovascular de Alta Complexidade (PNACAC) classifica os serviços de acordo com o tipo de atividade desenvolvida e estabelecendo quantitativo mínimo de produção anual: cirurgias cardiovasculares (180 procedimentos); procedimentos de cardiologia intervencionista (144); procedimentos endovasculares extracardíacos (120); cirurgias cardiovasculares pediátricas (120); cirurgias vasculares (90); e laboratório de eletrofisiologia (39). Esta política, no entanto, é baseada apenas no número de procedimentos e na capacidade instalada, não levando em consideração a real necessidade das diferentes regiões do país. O resultado é a distorção do sistema, com leitos para atendimento de doenças cardiovasculares escassos e mal distribuídos, e ainda um serviço pode ser considerado de alta complexidade cardiovascular sem a necessidade de contemplar todas as atividades da área acima citadas.

O levantamento dos autores,^5^ com dados de 2014, mostrou a existência de pouco mais de 3 mil leitos no Brasil destinados para a alta complexidade cardiovascular no SUS em 277 hospitais distribuídos irregularmente pelo Brasil. Apenas 9,6% destes possuem atendimento em cirurgia cardíaca pediátrica. Alguns estados como Tocantins não possuíam qualquer serviço com atendimento cardíaco pediátrico. Ainda, pouco mais de 20% destes hospitais pelo Brasil são públicos – apesar de boa parte dos privados serem filantrópicos (e, portanto, com vantagens tributárias), mesmo assim voltamos ao constante embate sobre o subfinancimento do SUS. São crianças que nascem com doenças cardíacas complexas, onde não é incomum a necessidade de várias intervenções ao longo da sua vida, realizadas por equipes multidisciplinares e diferentes especialidades.

Outro problema é a questão da equipe multidisciplinar. A evolução do diagnóstico e tratamento das cardiopatias congênitas exige não só formações específicas, mas também que estas diferentes especialidades atuem de forma complementar, integrada e algumas vezes simultânea. A PNACAC exige um número mínimo de procedimentos cirúrgicos para considerar o serviço como alta complexidade, mas não faz o mesmo quanto à realização de procedimentos igualmente importantes – muitos não possuem credenciamento, equipamento ou procedimentos em cardiologia intervencionista e/ou eletrofisiologia para aquela população concomitantemente ao serviço de cirurgia cardíaca, nem outras atividades de apoio como nutrição, exames complementares (tomografia e ressonância) ou fisioterapia especializada. Novos materiais e técnicas fazem com que a cardiologia intervencionista hoje seja uma alternativa importante e viável não somente para o diagnóstico, mas principalmente para o tratamento de muitas cardiopatias congênitas, podendo aumentar o número de pacientes atendidos e liberando o cirurgião cardíaco para realização de outros procedimentos que o cateterismo não consegue realizar.

Desta forma, retornando aos números acima, cruzando as informações de número de pacientes com cardiopatias congênitas que exigem tratamento ao nascimento (ou ao menos na faixa etária pediátrica) com o número de cirurgias, cateterismos e leitos realizados nesta população, temos certeza de que muitas destas crianças sequer têm seu diagnóstico confirmado, e mesmo que o tenham morram sem qualquer tipo de assistência. Ou às vezes evoluem para o óbito durante transferência para serviço de referência. A consequência é clara e imediata: são mortes “invisíveis”, que aparecem pouco nas estatísticas e na mídia, e por isto esquecidas e relegadas a um segundo plano. Entre as maiores causas de morte no Brasil e no mundo estão as doenças cardíacas.^6^ Mas estas, quando citadas, imediatamente são associadas com o infarto agudo do miocárdio, não às cardiopatias congênitas.

Em 2017 o Ministério da Saúde acenou com o início de uma mudança no lançamento do Plano Nacional de Assistência a Crianças com Cardiopatia Congênita,^7^ o qual integraria ações para o acesso ao diagnóstico, ao tratamento e à reabilitação de crianças cardiopatas. A meta inicial era ampliar em 30% o número de cirurgias feitas na rede pública de saúde. A portaria, publicada sob o número 1.727, deu um importante passo ao transferir o financiamento dos procedimentos cardíacos pediátricos para o Fundo de Ações Estratégicas e Compensação - FAEC). O resultado imediato foi o incremento de 8% no número de cirurgias cardíacas pediátricas um ano depois.^8^

Com todas as informações postas, fica óbvia a necessidade de se continuar avançando com o Plano Nacional de Assistência a Crianças com Cardiopatia Congênita, objetivando agora a abertura de leitos e a formação de novos serviços especializados no diagnóstico e tratamento daquelas doenças. As malformações do aparelho cardiovascular são causa importante de mortalidade nos recém-nascidos e infantes e precisam ser mais valorizadas pelo poder público. Não há como se falar em sistema de atendimento universalizado quando alguns são mais iguais do que outros; quando nem todos que precisam tem acesso sequer ao diagnóstico, muito menos ao tratamento. E quando conseguem ser tratados, mas ainda assim o SUS não possibilita ofertar as mesmas possibilidades de materiais ou procedimentos da iniciativa privada. Estes fatores, aliados a uma extensa formação de profissionais especializados e ao subfinanciamento do sistema, deixam claro que somente atitudes mais objetivas por parte de governos federal, estaduais e municipais irão mudar este panorama.
