# Supplementary material for: Panorama e Estratégias no Diagnóstico e Tratamento de Cardiopatias Congênitas no Brasil
Source: Arq Bras Cardiol. 2020 Dec 1;115(6):1176–7. [Article in Portuguese] doi: 10.36660/abc.20200680 (PMC8133732; doi:10.36660/abc.20200680)
Supplement: Translation

# Translation: Outlook and Perspectives in Diagnosis and Treatment of Congenital Heart Diseases in Brazil

**Authors:** Fabio Augusto Selig

**Affiliations:** Brazil CONCEPT - Centro de Cardiopatias Congênitas e Estruturais do Paraná, Curitiba, PR - Brazil

**Keywords:** Constitution and Bylaws, Unified Health System, Heart Defects Congenital/diagnostic, Healthcare Financing

The 1988 Brazilian Constitution, also called the “Citizen Constitution”, established universal access to health when, in its article 196, it stated that this is the right of all citizens and the Government’s responsibility. Just over a decade later, the Brazilian Unified Health System (SUS, *Sistema Único de Saúde* ) was created, which, in order to respect the principles of universality, integrality and equality established in the Magna Carta, laid down guidelines for decentralization, comprehensive care and popular participation.

Very much inspired at the time on the English health system, SUS is undoubtedly one of the largest public health systems worldwide, responsible for approximately 190 million people, 80% of whom depend exclusively on it to take care of their health, as they do not have access to the private health system. Still, even among those with access to the private system, the use of the public health system is constitutionally guaranteed, without limits or restrictions.

SUS financing is undoubtedly one of the biggest challenges faced during its 20 years of existence, because although the funds are in theory guaranteed by Constitutional Amendment Number 29 and by several other legal provisions, the breadth of its coverage makes it impossible to be constantly updated in coverage and paid amounts. In fact, despite being theoretically a universal and integral system, the consequence of financing difficulties is that the public system offers less comprehensive preventive, diagnostic and therapeutic resources than the private system, which has created a huge gap between the two systems over time. Therefore, it becomes essential to develop clear public health policies to manage these resources and, probably, at some point a broad re-discussion of the role of the public and private systems in serving the population will have to take place.

In this issue, we will have the opportunity to read an important article.^1^In a clear, objective and technical way, it demonstrates with concrete numbers obtained from the official SUS database the importance that congenital heart diseases have in the mortality of individuals younger than 20 years of age, and especially in children under 1 year. The study shows that this universal coverage is not reflected in real life when it comes to this group of diseases.

The incidence of congenital heart disease in the general population is 8 in every 1,000 live births.^2^Using the Brazilian birth rate,^3^we would have approximately 30,000 new births of children with heart disease. In at least half of these newborns, heart disease shows a good evolution, with spontaneous cure or non-severe evolution, to the point that the diagnosis is made only in adulthood.^4^Therefore, still approximately, we would have 15,000 newborns who would annually require some type of treatment, often surgical or through interventional cardiology, and many of them soon after birth or during the first year of life.

Keeping this reasoning in mind, official data indicate the scarce number of high cardiovascular complexity hospitals that provide care to SUS patients in Brazil.^5^

The National Policy for High Complexity Cardiovascular Care (PNACAC, *Política Nacional de Atenção Cardiovascular de Alta Complexidade* ) classifies the services according to the type of activity developed and establishes a minimum quantity of annual production: cardiovascular surgeries (180 procedures); interventional cardiology procedures (144); extracardiac endovascular procedures (120); pediatric cardiovascular surgeries (120); vascular surgeries (90); and electrophysiology laboratory procedures (39). This policy, however, is based only on the number of procedures and the established capacity, not taking into account the real need of the different regions in the country. The result is the distortion of the system, with scarce and poorly distributed beds for the care of cardiovascular diseases and, also, a service can be considered of high cardiovascular complexity without the need to encompass all the area activities mentioned above.

The authors’ survey,^5^using data from 2014, showed the existence of a little over 3,000 beds in Brazil reserved for High Cardiovascular Complexity in SUS in the 277 hospitals irregularly distributed throughout Brazil. Only 9.6% of them have assistance in pediatric cardiac surgery. Some states, such as Tocantins, do not have a service with pediatric cardiac care. Still, just over 20% of these hospitals in Brazil are public – although most of the private ones are philanthropic (and therefore have tax advantages) – even so we are back to the constant struggle over the underfunding of the Unified Health System. These are children born with complex heart disease, where it is not uncommon to need several interventions throughout life, carried out by multidisciplinary teams and different specialties.

Another problem is the issue of the multidisciplinary team. The evolution of the diagnosis and treatment of congenital heart diseases requires not only specific training, but also that these different specialties act in a complementary, integrated and sometimes simultaneous way. The PNACAC requires a minimum number of surgical procedures to consider the service as high complexity, but it does not do the same for the performance of equally important procedures – many do not have accreditation, equipment or procedures in interventional cardiology and / or electrophysiology for that population concurrently with cardiac surgery service, nor other support activities such as nutrition, complementary exams (computed tomography and MRI) or specialized physiotherapy. New materials and techniques make interventional cardiology today an important and viable alternative not only for the diagnosis, but mainly for the treatment of many congenital heart diseases, which can increase the number of patients that are treated, thus freeing the cardiac surgeon to perform other procedures that the catheterization team cannot do.

Thus, returning to the numbers above, crossing the information on the number of patients with congenital heart disease requiring treatment at birth (or at least in the pediatric age group) with the number of performed surgeries and catheterizations and beds available for this population, we are sure that many of these children do not even have their diagnosis confirmed, and even if they have, they might die without any assistance. Or sometimes they die during transfer to a referral service. The consequence is clear and immediate: they are “invisible” deaths, which appear little in the statistics and in the media and are therefore forgotten and relegated to a secondary level. Among the most important causes of death in Brazil and worldwide are heart diseases.^6^But these deaths, when mentioned, are immediately associated with Acute Myocardial Infarction, not with congenital heart diseases.

In 2017, the Ministry of Health signaled the starting of change when the National Assistance Plan for Children with Congenital Heart Disease ( *Plano Nacional de Assistance a Crianças com Cardiopatia Congênita* )^7^was launched, which would integrate actions for access to diagnosis, treatment and rehabilitation of children with heart disease. The initial goal was to increase the number of surgeries performed in the public health system by 30%. The Ordinance, published under number 1,727, took an important step in transferring funding for pediatric cardiac procedures to the Fund for Strategic Actions and Compensation (FAEC, *Fundo de Ações Estratégicas e Compensação* ). The immediate result was an 8% increase in the number of pediatric cardiac surgeries a year later.^8^

Considering all these data, it is obvious that there is an urgent need to move forward with the National Assistance Plan for Children with Congenital Heart Disease, now aiming to opening beds and training new specialized services in the diagnosis and treatment of those diseases. Cardiovascular system malformations are an important cause of mortality in newborns and infants and need more attention by the government. It is impossible to talk about a universal health care system when some are more equal than others; when not everyone in need has access to diagnosis nor adequate treatment. And when they are managed to be treated, still SUS does not offer the same possibilities of materials or procedures as the private sector does. These factors, together with an extensive training of specialized professionals and the underfunding of the system, make it clear that only more objective attitudes on the part of the federal, state and municipal governments will change this scenario.
